# Towards the Clinical Evaluation of the Luteal Phase in Fertile Women: A Preliminary Study of Normative Urinary Hormone Profiles

**DOI:** 10.3389/fpubh.2018.00147

**Published:** 2018-05-31

**Authors:** María Elena Alliende, José Antonio Arraztoa, Ulises Guajardo, Fernando Mellado

**Affiliations:** ^1^Programa de Cuidado y Estudio de la Fertilidad (PROCEF), Departamento de Obstetricia, Ginecología y Biología de la Reproducción, Universidad de los Andes, Santiago, Chile; ^2^Facultad de Medicina, Universidad de los Andes, Santiago, Chile

**Keywords:** luteal phase, luteal phase deficiency, luteinizing hormone, estrone glucuronide, pregnandiol glucuronide, ovulation detection, cervical mucus, Natural Family Planning

## Abstract

**Objective:** To describe and evaluate urinary hormone profiles in the luteal phase.

**Setting and Patients:** Twenty-five healthy fertile women, with regular ovulatory pattern cycles as assessed by temperature and cervical mucus, at a university based center.

**Methods:** Daily urinary hormonal assessment of luteinizing hormone, estrone glucuronide, and pregnanediol glucuronide. This was done during 3 or more cycles, with 78 completed cycles. Samples were analyzed by both crude levels and levels adjusted for the hormone excretion rate. Correlation between measured parameters (LH surge, vulvar mucus) was assessed with regard to their ability to detect presumed ovulation.

**Results:** An upper, middle, and lower tercile range for the main urinary reproductive hormones was determined and a classification system of zones proposed, considering profiles over or under the 10th percentile. Adjustment for the urine excretion rate proved useful for interpreting individual samples; this was less necessary with multiple samples over time where trends could be determined. This serial evaluation, in at least two cycles, lowered the possibility of finding an isolated luteal phase defect and helped identify the recurrence of such. Vulvar mucus findings performed well in determining the timing of ovulation. Despite the proven fertility of the study population, lower luteal phase hormones were detected in both an isolated and, in some situations, recurrent manner.

**Conclusion:** A feasible method is proposed to accurately, thoroughly and reproducibly study the luteal phase in order to evaluate and treat identified abnormalities in a properly timed, restorative manner. This preliminary study provides the basis for future research, correlating urinary hormones with clinical findings, particularly those of luteal phase defects.

## Introduction

The role of a physiological menstrual cycle is to guarantee proper reproduction. Since luteal function develops as a result of all the preceding processes of the cycle, its evaluation seems to be an appropriate means in characterizing the entire cycle. Although the diagnosis of luteal phase defects (LPD) has been described convincingly in a research setting, it remains a controversial clinical entity (1, 2). Luteal phase impairment in natural cycles is a plausible cause of infertility and pregnancy loss. Thus, it is critical for the clinician to understand and recognize deficient luteal phases. Different factors may vary from cycle to cycle, making it important to determine that a defect is repetitive (3).

Confusion surrounding the evaluation of luteal phase quality is the result of inconsistent and unreliable diagnostic criteria. The use of low luteal phase serum progesterone as a diagnostic tool proves challenging because of the pulsatile release of progesterone from the corpus luteum, following the pulsatile release of luteinizing hormone (LH) from the pituitary. Serum progesterone levels can fluctuate widely in healthy subjects. With that being said, random samples of serum progesterone levels, whether single or multiple, may not be helpful in the diagnosis. The secretory pattern of progesterone results in wide confidence limits such that samples from individuals cannot be compared to normal samples in a useful manner. Despite this, isolated serum progesterone concentrations are still used to characterize the luteal phase. Daily serum luteal progesterone would be more accurate; however, it is clinically impractical. No current method for the diagnosis of luteal phase quality appears to be usefully applied in the clinical setting. Research should concentrate on a precise diagnostic test. The use of urinary pregnanediol glucuronide (PG) level has been suggested to minimize progesterone fluctuations (4).

Ovarian hormonal activity can be accurately monitored with early morning urine samples (5, 6). Serum LH, estradiol and progesterone correlate very well with urinary LH (7–9), estrone glucuronide (EG), and PG [(10–14)]. Lately serum LH, estradiol and progesterone have been confirmed to correspond well with urinary LH, EG, and PG (15).

These specimens are simple to collect from a small sample of early morning urine, can be stored in a freezer at home and are stable to transport. All of these factors make it clinically practical to perform a serial urinary reproductive hormonal profile during one or more cycles. Urinary concentration correction has been claimed as unnecessary (15). However, for clinical application, adjustment for concentration or urinary excretion rate could be desirable to improve accuracy on an individual basis (16).

Adequate identification of ovulation in order to properly evaluate the luteal phase is also a problematic matter. Although seldom performed, this can be done presuming ovulation through self-perceived cervical mucus changes at the vulva. It requires some degree of training and compliance, but if this issue can be overcome, this method can be very reliable (17, 18). In research settings, with a retrospective approach, the rise of LH over a baseline has been found to closely correlate with the timing of ovulation (15). In clinical settings, with a prospective approach, the use of home kits that detect threshold urinary LH rise to target the luteal phase has become main stream. However, a recent paper points out the inaccuracy of using these threshold LH tests by themselves to detect ovulation (19), even though, they could be utilized along with PG rise threshold in-home tests or with the peak mucus sign.

This study intends to offer a clinical evaluation of the luteal phase providing a preliminary reference range for the major urinary reproductive hormones, obtained from healthy fertile women cycling regularly.

## Materials and methods

This was a secondary analysis of data from a World Health Organization (WHO) sponsored study (HRP # 87904) (20) in a university based Natural Family Planning Center (Unidad de Métodos Naturales, Hospital Clínico, Universidad de Chile). The initial study was approved by the Medical Faculty Ethics Committee at the University of Chile. Each woman gave written informed consent.

### Population

The study was done in 25 white, healthy, proven-fertile women. All were users of Natural Family Planning [mean and median age: 30, standard deviation (*SD*) ± 4, and range: 24–37 years]. They identified their fertile period by perceiving changes in their cervicovaginal fluid at the vulva, using a local variant of the Ovulation Method (21). They also identified a shift in their basal body temperature (BBT).

All had regular menstrual cycles, 25–35 days in the previous six cycles. The study cycles lasted a mean and median of 28 days (*SD* ±2.5, range 23–35 days). All the previous cycles, and also the study cycles had records with a potentially fertile period that included ovulatory mucus patterns and biphasic BBT graphs. No significant premenstrual spotting was observed.

The subjects were not taking any form of hormonal contraception in the previous six cycles and had not been breastfeeding in the last 6 months. They did not perform vigorous exercise and they had a normal body mass. No subject had any history or evidence of liver or kidney disease, or dysfunction (which might affect the urinary excretion of hormone metabolites). The subjects furthermore did not possess any form of chronic drug therapy.

### Urinary hormone assays

A small early morning urine sample, timed and measured for volume, was obtained daily from the 25 women during 3 or more cycles. This entailed recording the time of the last urination and measuring the time and volume of the early morning urine, from which they took a sample to store for a limited period in their home freezer. All the samples came from early morning urine of more than 100cc and at time intervals >3 h (to effectively measure excretion rates). The frozen urine samples were collected fortnightly from each volunteer and taken to the laboratory for analysis, after completion of the study cycles (Laboratorio de Endocrinología y Biología Reproductiva Hospital Clínico, Universidad de Chile).

Estrone-3-glucuronide: E_1_-3-G, (EG); pregnanediol-3 alpha-glucuronide: Pd-3α-G, (PG), and luteinizing hormone (LH) were measured by non-competitive radioimmunoassays. The reagents and assay protocols were supplied by the Matched Reagents Programme of the WHO (Queen Charlottes and Chelsea Hospitals, Goldhawk Road, London, UK) and the local laboratory participated in a program of external quality assessment. Aliquots of each urine sample were saved for further assay if deemed necessary. All the urine specimens from the same subject were assayed in one batch. When this was not technically possible, all samples from the same cycle were assayed in one batch.

When reporting the results without the hormone urine excretion rate (assumed per liter), numbers were recorded in standard international units: nmol/L for EG, IU/L for LH, and μmol/L for PG. To calculate the hormone urine excretion rate, assay results in units per liter were multiplied by the total volume of the early morning urine from which the sample was collected, in liters. The product was divided by the time elapsed since the last urine void in hours. The outcome, in units per liter per hour, was multiplied by 24. Results then are expressed as units per liter without considering the actual excretion rate and in units per liter per 24 h when considering the actual hormone urine excretion rate. To compare with other studies which express their results in other units, PG is converted from μg/ml to standard unit μmol/L, multiplying by 0.32 (vice versa by 3.12). EG is converted from ng/ml to standard unit nmol/L, multiplying by 2.24 (vice versa by 0.44). We have to consider that excretion rates differ from concentration, usually adjusted for creatinine.

A total of 82 cycles were gathered. Four cycles with incomplete urinary samples were excluded. Seventy eight cycles were completed. All the women provided at least 3 complete cycles. In each cycle, a urinary sample for hormonal study was obtained almost every day. Only 5 isolated luteal phase samples were missing.

### Definitions

LH and PG rise: details of how to determine this are in Supplement 1.

LH presumed ovulation: the day after the LH rise.

Mucus peak day: the last day of lubricated vulvar sensation and/or any degree of clear, bloody or stretchy mucus (≥2.5 cm) detected at the vulva.

Mucus presumed ovulation: the mucus peak day.

“Very poor” follicular mucus: fertile period is only perceived but no mucus is detected at the vulva, or for up to one day, opaque white mucus that stretches ≤1 cm is recognized.

Luteal phase: the luteal phase begins the day after the presumed ovulation day and ends the day before the onset of the next menstruation.

The luteal phase was divided into an initial, medial, and final zones.

The luteal phase hormonal profile was divided as greater and ≤ the 10th percentile.

More details of this procedure are provided in Supplement 2.

### Statistical analysis

Chi square tests were used to study the association between follicular mucus perception and hormonal profile during the late follicular or luteal phase in menstrual cycles. Follicular mucus perception was considered “very poor” or not “very poor”, and luteal phase as greater and ≤10th percentile.

Sensitivity and specificity for luteal PG not adjusted for excretion rate was calculated in each luteal day sample considering PG ≤ 5th percentile adjusted for the excretion rate as the gold standard (true positive).

All the statistical analysis and graphs were performed using Microsoft Excel 2010. A *p*-value under 0.05 was considered for statistical significance.

## Results

**Figure 1:**
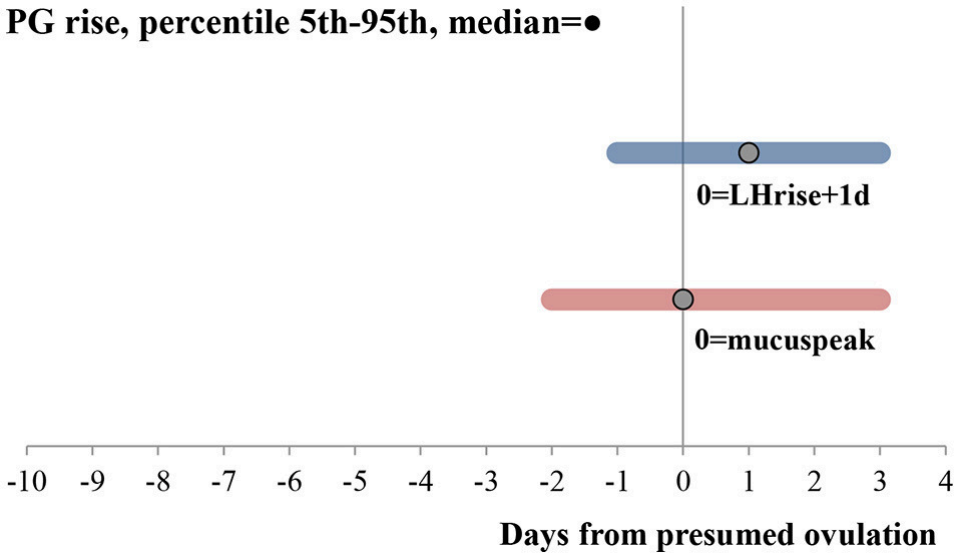
PG rise relative to ovulation assessed through LH rise and mucus peak. Bars go from the 5th to 95th percentiles. Median is shown with a dot. *N* = 78 cycles. Both hormonal rises were calculated according to the description in the methods. PG and LH values were adjusted for urine excretion rates.

All the cycles showed an LH and PG rise. Details of the LH rise, mucus peak, PG rise and luteal phase length are shown in Table 1. Within 7 cycles after an initial PG rise, before the definite PG rise, PG fluctuated without reaching the threshold value defined for the cycle for 3 consecutive days. The median and 5th to 95th percentiles of the PG rise, relative to presumed ovulation indicated by the mucus peak and LH rise are shown in Figure 1.

**Table 1:** Relationship between different criteria to define the luteal phase.

	**Cycle day**	Days relative to LH rise+1 day (adjusted^*^)	**Days relative to mucus peak**	Days relative to PG rise (adjusted^*^)	**Luteal phase length (days)**
Mucus Peak	16.4 ± 2.7 (10 to 26)	0.9 ± 1.1 (−2 to 4)	X	−0.3 ± 1.5 (−4 to 4)	12.2 ± 1.5 (9 to 16)
LH rise+1day (adjusted^*^)	15.5 ± 2.4 (11 to 23)	X	−0.9 ± 1.1 (−4 to 2)	−1.1 ± 1.2 (−4 to 2)	13.1 ± 1.2 (10 to 17)
LH rise+1day (not adjusted^*^)	15.5 ± 2.6 (11 to 25)	0.05 ± 0.7 (−1 to 3)	−0.8 ± 1.5 (−4 to 2)	−1.1 ± 1.3 (−4 to 2)	13.0 ± 1.4 (9 to 17)
PG rise (adjusted^*^)	16.6 ± 2.5 (11 to 23)	1.1 ± 1.2 (−2 to 4)	0.3 ± 1.5 (−4 to 4)	X	11.9 ± 1.5 (9 to 17)

*Luteal phase begins the day after the presumed ovulation (day 0). Data are shown as mean ± SD (range)*.

* *Adjusted for urine excretion rate*.

The profile of the luteal phase detected per woman, after considering 3 or more cycles, was similar when the luteal phase was located through presumed ovulation with LH or mucus. Results will be shown only considering presumed LH ovulation.

### Biomarkers

All the cycles had an ovulatory mucus pattern associated with a biphasic BBT, without a short luteal phase or prolonged premenstrual spotting. Different details in biomarkers were explored in search of an association with the hormonal profile of the luteal phase. A significant relationship could only be established between “very poor” follicular mucus perception and luteal EG levels ≤ 10th percentile (*p* < 0.001). No association was found between follicular mucus perception and luteal PG level (*p* = 0.4).

### PG profile

**Figure 2:**
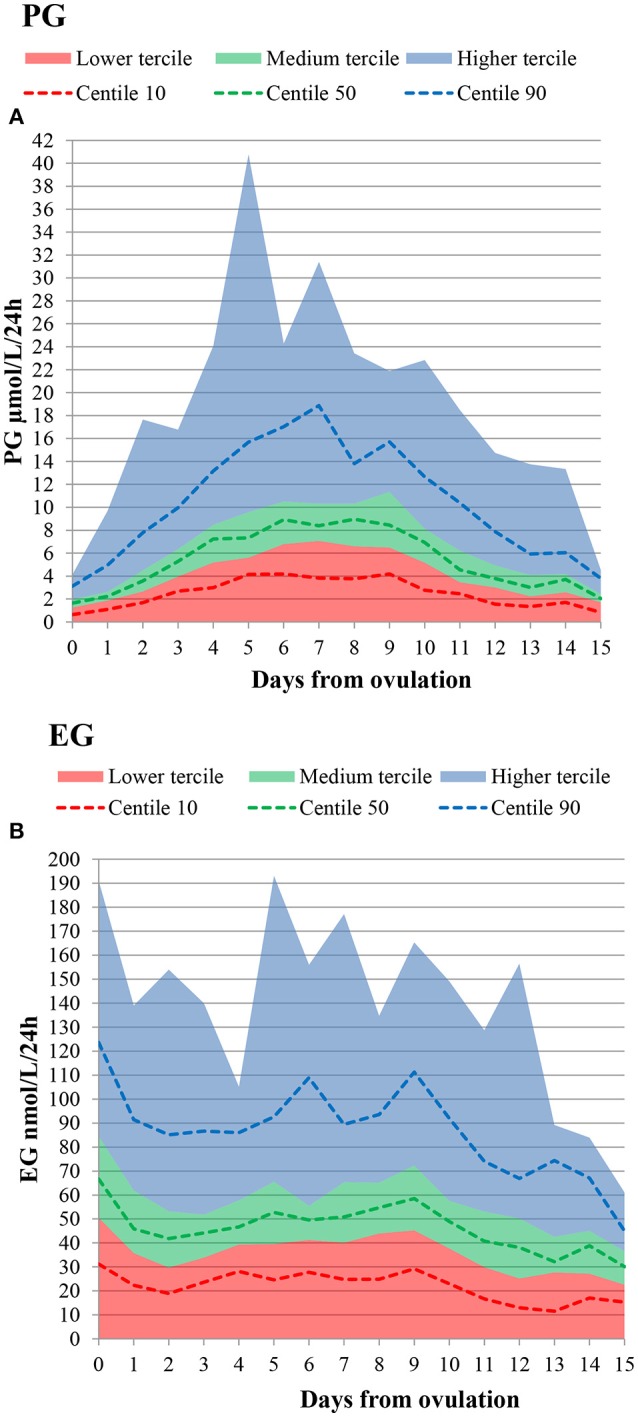
Daily lower, medium and higher tercile hormone ranges, as well as 10th, 50th, and 90th percentiles. Presumed ovulation (LH rise+1d) is day 0. **(A)** PG **(B)** EG.

**Figure 3:**
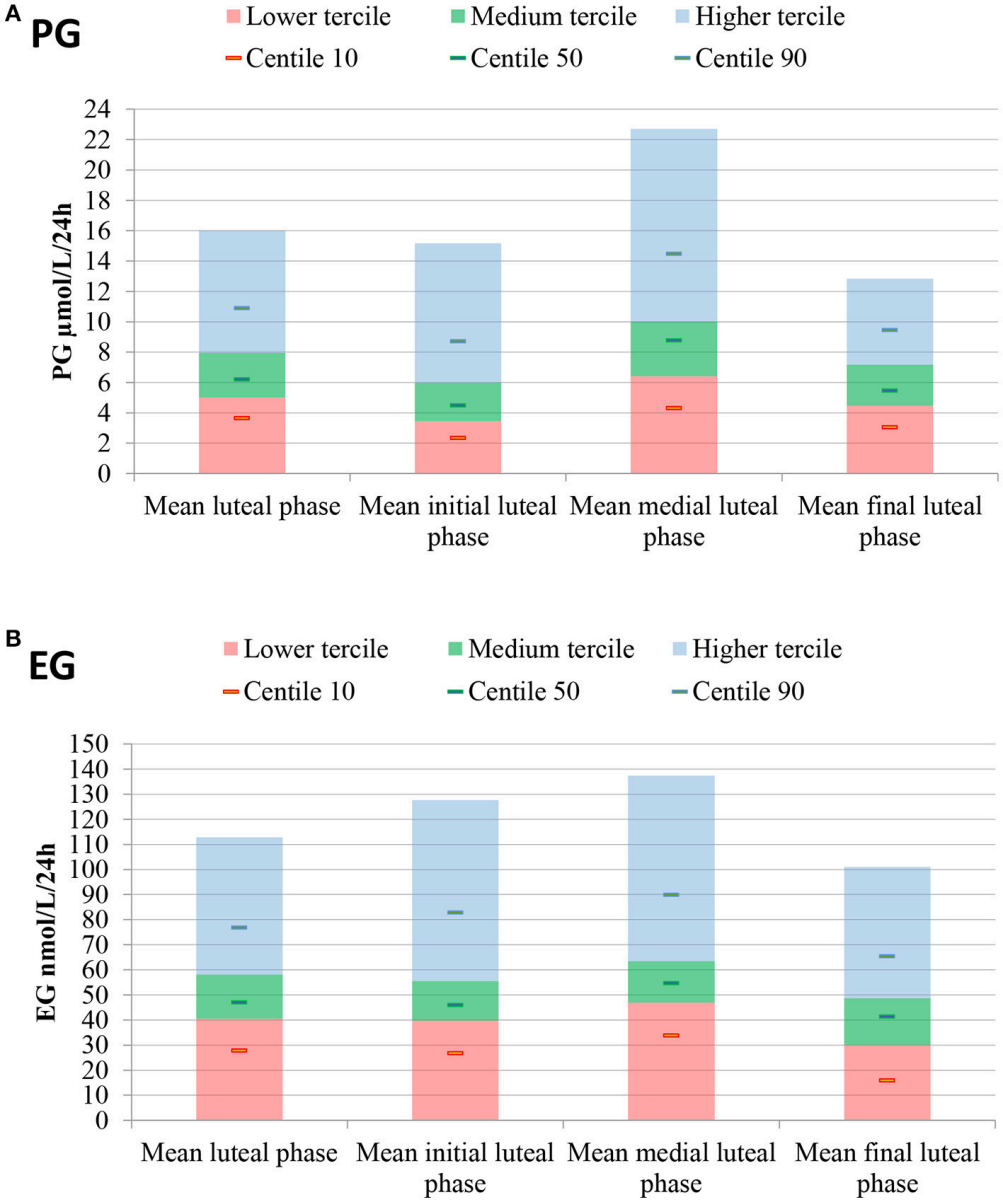
Mean luteal, lower, medium and higher tercile hormone ranges, as well as 10th, 50th, and 90th percentiles. Luteal hormones are also presented by zone. **(A)** PG, **(B)** EG. Luteal phase determined through LH presumed ovulation (LH rise+1d).

The daily luteal ranges of urinary PG with respect to LH presumed ovulation day are provided in Table 2. The ranges are also shown in Figure 2A. PG per luteal zone is shown in Figure 3A.

**Table 2:** Daily values of PG and EG levels adjusted for urine excretion rate relative to presumed ovulation (LH rise +1day).

	**PG** μmol/L/24h (adjusted^*^)				EG nmol/L/24h (adjusted^*^)			
**Days from ovulation**	***N***	**Median (10–90 centiles)**	**Low tercile range**	**Medium tercile range**	**High tercile range**	**Median (10–90 centiles)**	**Low tercile range**	**Medium tercile range**	**High tercile range**
0	78	1.7 (0.6–3.1)	≤1.2	>1.2– ≤ 1.8	>1.8–3.7	66.6 (31.2–123.6)	≤50.5	>50.5 ≤ 84.5	>84.5–191.0
1	78	2.2 (1.1–5.0)	≤1.8	>1.8– ≤ 2.5	>2.5–9.6	45.9 (22.4–91.4)	≤35.8	>35.8– ≤ 61.9	>61.9–139.0
2	78	3.6 (1.7–7.8)	≤2.6	>2.6– ≤ 4.3	>4.3–17.2	41.8 (18.9–85.1)	≤29.7	>29.7– ≤ 53.3	>53.3–154.0
3	76	5.3 (2.7–10.0)	≤3.9	>3.9– ≤ 6.0	>6.0–16.6	44.2 (23.6–86.6)	≤33.9	>33.9– ≤ 51.9	>51.9–140.0
4	78	7.2 (3.0–13.2)	≤4.9	>4.9– ≤ 8.0	>8.0–23.8	46.8 (28.1–86.0)	≤39.3	>39.3– ≤ 57.8	>57.8–105.1
5	77	7.3 (4.2–15.7)	≤5.5	>5.5– ≤ 8.9	>8.9–39.8	52.8 (24.7–92.6)	≤39.6	>39.6– ≤ 65.6	>65.6–193.1
6	78	8.9 (4.2–17.0	≤6.6	>6.6– ≤ 10.3	>10.3–24.1	49.6 (27.8–108.9)	≤41.3	>41.3– ≤ 55.5	>55.5–156.0
7	77	8.4 (3.8–18.9)	≤6.9	>6.9– ≤ 9.8	>9.8–30.9	50.9 (24.8–89.4)	≤40.1	>40.1– ≤ 65.4	>65.4–177.1
8	77	9.0 (3.8–13.8)	≤6.5	>6.5– ≤ 10.0	>10.0–23.3	54.8 (24.9–93.6)	≤44.0	>44.4– ≤ 65.1	>65.1–134.7
9	78	8.4 (4.2–15.7)	≤6.3	>6.3– ≤ 10.7	>10.7–21.8	58.5 (29.2–111.2)	≤45.2	>45.2– ≤ 72.3	>72.3–165.3
10	78	6.9 (2.8–12.7)	≤5.1	>5.1– ≤ 8.0	>8.0–22.5	49.0 (23.0–92.0)	≤37.7	>37.7– ≤ 57.6	>57.6–149.1
11	77	4.6 (2.5–10.4)	≤3.4	>3.4– ≤ 6.2	>6.2–18.1	40.8 (16.7–74.0)	≤29.9	>29.9– ≤ 53.1	>53.1–128.7
12	73	3.8 (1.6–7.9)	≤2.9	>2.9– ≤ 4.7	>4.7–14.6	38.1 (13.0–66.9)	≤25.2	>25.2– ≤ 50.2	>50.2–156.4
13	49	3.0 (1.3–5.9)	≤2.2	>2.2– ≤ 4.1	>4.1–13.6	32.2 (11.6–74.4)	≤27.7	>25.2– ≤ 42.6	>42.6–89.2
14	24	3.7 (1.7–6.0)	≤2.6	>2.6– ≤ 4.2	>4.2–13.2	38.9 (17.1–67.1)	≤27.2	>27.2– ≤ 45.1	>45.1–83.9
15	9	2.0 (0.8–3.8)	≤1.7	>1.7– ≤ 2.4	>2.4–4.5	30.1 (15.4–45.0)	≤22.6	>27.2– ≤ 36.6	>36.6–61.0

*Presumed ovulation is day 0. Data shown as median (10th and 90th percentiles) and tercile ranges. Number of cycle samples are displayed per day*.

* *Adjusted for urine excretion rate*.

Detail per woman and per cycle of luteal phase PG profile is shown in Table 3.

**Table 3:** Luteal phase hormonal profile per woman **(A)** and per cycle **(B)**.

**(A)**		
**Luteal profile per woman**	**EG**	**PG**
	***N*%**	***N*%**
>10th percentile	16 64	16 64
Exclusively ≤ 10th percentile	5 20	4 16
Recurrent ≤ 10th percentile	4 16	5 20
**(B)**		
**Luteal profile per cycle**	**EG**	**PG**
	***N*****%**	***N*****%**
> 10th percentile	61 78	61 78
“Partially low” ≤ 10th percentile	5 7	8 10
“Low” ≤ 10th percentile	12 15	9 12

*N = 25 women, ≥3 cycles each, N = 78 cycles. Recurrent is on two or more cycles. “Partially low” and “Low” description in Supplement 2*.

Considering only the cycles with luteal PG ≤ 10th percentile, approximately half did not reach the 10th percentile in the initial zone (53%), in the middle zone (53%), and/or in the final luteal zone (47%).

Lowering the threshold, 16% of women exclusively (for 1 cycle) and 8% of women recurrently (for ≥2 cycles) did not reach the 5th percentile.

All the cycles reached a maximum luteal PG level over 3.2 μmol/L/24 h (10 μg/ml).

### EG profile

The daily luteal EG ranges are shown in Table 2 and Figure 2B. EG per luteal zone is shown in Figure 3B.

Detail per women and per cycle of luteal phase EG profile is shown in Table 3.

Considering only the cycles with luteal EG ≤ 10th percentile, about half did not reach the 10th percentile, in the initial (47%), medial (47%), and/or final (47%) luteal zone.

Lowering the threshold, 12% of women exclusively (for 1 cycle) and 12% of women recurrently (for ≥ 2 cycles) did not reach the 5th percentile.

### Concurrent profile of luteal EG and PG

In 4 cycles from 3 women, there were levels of PG and EG ≤ 10th percentile simultaneously. This happened recurrently (for ≥ 2 cycles) in only one woman. Considering a lower threshold, a single woman in one cycle had luteal EG and PG simultaneously below the 5th percentile.

### Urine excretion rate adjustment to determine hormonal profile

**Figure 4:**
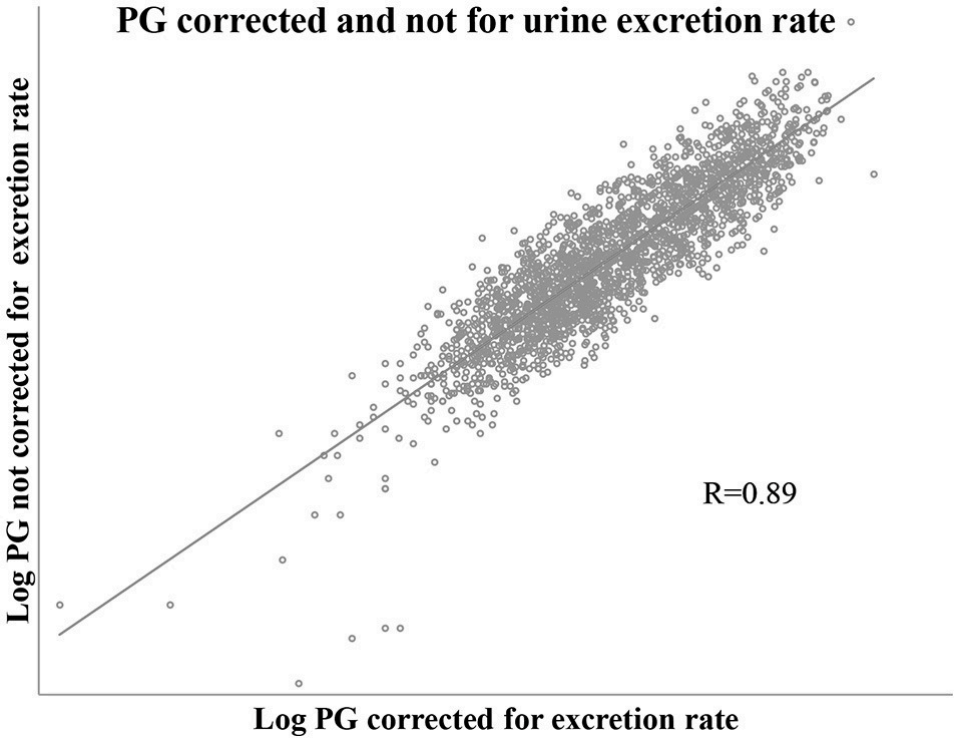
Scatter plot showing correlation of PG values corrected and not corrected for urine excretion rate. Includes one pair of PG values for each of all study cycle days, *n* = 2,131 pair of values.

The values of PG adjusted for urine excretion rate (volume, time) had a correlation of 0.89 with the correspondent values that did not consider urine excretion rate. This is shown in Figure 4.

Despite this high correlation, considering each luteal PG sample (*n* = 987), and as true positive PG adjusted for excretion rate ≤ 5th percentile; those values not adjusted for excretion rate had a sensitivity of 0.55 and a specificity of 0.95.

### LH threshold rise to detect ovulation

This information has been eliminated because during the peer review process a more complete article addressing these issues has been published, with similar findings but better referenced to ovulation detected by ultrasound (19).

As a circumstantial finding, 5 of 6 women with poor luteal PG, reported having had newborn(s) of <2.5 kg in their obstetrical history. This type of association was not reported in women with poor luteal EG.

## Discussion

The preliminary normative urinary luteal profiles of estrone-3-glucuronide and pregnanediol-3-glucuronide indexed to urinary LH have been presented in 78 normal cycles obtained from 25 fertile women. Correlation to the peak of cervical mucus had similar results.

### EG and PG ranges

The ranges of EG and PG obtained seem to be higher than those found recently (22). This can be due to a different type of population: all the women in our study had proven fertility, vs. the 57.5% of the women who did not have proven fertility in the mentioned study. The difference in hormonal assays should also be considered. In addition, the values are expressed taking into account urinary concentration adjusted for creatinine; instead of adjustment through excretion rates, as was done here. Moreover, the fertility status of women may have changed over time.

Compared to what has been previously described dividing the luteal phase with a threshold of 3.2 μmol/L24 h (23), every cycle in this study has overpassed this threshold, vs. only 92% of the cycles in the cited study. A shorter luteinization, longer progestation, and shorter lutetolysis processes have been found herein (not shown). Both studies were originally conducted around the same time. The difference could again be due to a more selected population in our study. The dissimilarity in hormonal assays and the consideration of urinary concentration, instead of excretion rates could play a role as well.

### Assays

On the other hand, in this study, the immunoassay ranges for EG and PG appear to be lower than those obtained with the Home Ovarian Monitor enzyme immunoassays (24). We previously found higher hormone ranges in a study with EG and PG measured using the Ovarian Monitor, referenced with ultrasound ovulation in 30 cycles from 15 healthy fertile women (18, 25). These hormone ranges have not been shown in the cited papers. The Ovarian Monitor assays also consider actual hormone excretion rates, as herein.

Higher EG ranges have also been found when comparing the Ovarian Monitor enzyme immunoassays with WHO monitored radioimmunoassays (26), similar to those done in the present study.

### Issues related to urine samples

Urine concentration and actual hormone excretion rates concerns can be attenuated with a serial hormonal profile, during more than one cycle. Proper and regular liquid intake could also help, within a sequenced sampling. Urine concentration or excretion rate adjustment would be recommendable in isolated samples for more accuracy and to avoid persistent concentrated (false –) or diluted (false +) urine.

In-home tests to screen for PG levels can now be developed with higher thresholds, to identify decreased luteal hormone ranges. This could also be done for EG. A feasible approach to precisely evaluate the luteal phase would be to collect at-home early morning urine samples for a serial luteal hormonal profile, then transport the specimens for analysis to a lab. In-home screening tests to identify poor hormone ranges could also be helpful for treatment evaluation.

### EG and PG profile

In this carefully chosen fertile population without known clinical LPDs, luteal phases with PG and EG mostly over the 10th percentile have been found, despite isolated luteal phase findings in some women with levels ≤ 10th percentile. Indeed, in 16 to 20% of the women, there were recurrent (for ≥2 cycles) luteal phases ≤ 10th percentile. In 8–12% of the women there were recurrent luteal phases under the 5th percentile.

The cycle biomarkers did not demonstrate appreciable changes in some cases with luteal PG repeatedly below the 10th or 5th percentile. A significant relationship could only be established between “very poor” follicular mucus perception and luteal EG levels ≤ 10th percentile. If recurrent, this could have clinical implications that would deserve additional study.

### PG profile

Many studies that have defined luteal progesterone or PG thresholds have found healthy cycling women with deficient luteal phases [(24, 27, 28)]. The deficient luteal phase in women with regular cycles has been associated with low bone mass (29), and higher cardiovascular risk (30). Studying two cycles, recurrent suboptimal serum progesterone was found in 2% of the luteal phases in healthy regularly menstruating women, not all with proven fertility (31). This is fewer than the 8% found here with recurrent luteal PG below the 5th percentile. We have to consider that the mentioned study took only three luteal samples (early, mid, and late luteal phase) and assumed values in between. They also admit that the fertility monitors and/or the increase in LH used to presume ovulation may have led to misclassification of the luteal phase. In addition, they could have used a serum luteal progesterone threshold equivalent to lower urinary PG levels to define suboptimals. Altogether, these reasons could explain the differences found. In another publication (32), based on the same mentioned study (31) of healthy regularly menstruating women, those with sporadic anovulation tended to have lower estrogen, progesterone and peak LH levels in their ovulatory cycles.

These could signal hidden recurrent ovulary dysfunction (33), which could be the case of the women in our study that presented with recurrent luteal phases with hormones ≤ 10th percentile.

### EG profile

The luteal EG ≤ 10th percentile that was found in some cycles, recurrently in a few women, has already been similarly described with serial luteal serum estrogen (34).

### Concurrent EG and PG profile

Although there was not a strong association between simultaneous luteal EG and PG ≤ 10th percentile, in more cases there could be an association of decreased levels of both hormones concomitantly, taking into account higher thresholds, considering lower tercile ranges.

Concerning the clinical biomarkers, in this selected population, only “very poor” follicular mucus perception showed a significant relationship with poor luteal EG; but not with luteal PG.

### Luteal phase and pregnancy outcome

This association has been described by Hilgers (35). Progesterone levels at conception have been found linearly associated with miscarriage, preterm birth, intrauterine growth retardation, and eutrophic term birth (36).

Luteal estrogen deserves more study. Up until now luteal progesterone has been the focus of attention, deemed as clinically more relevant. The best results obtained in the case of repeated abortion (36), when only the follicular phase was stimulated to optimize the luteal phase -producing higher estrogen and progesterone- suggests the importance of luteal estrogen. Corpus luteum estradiol metabolites could play a role in angiogenesis and its functional lifespan and regression (37).

### Proper identification of the luteal phase

The goal is to effectively locate ovulation, and consequently the luteal phase, in a clinical environment.

### LH and PG in-home threshold tests

The urinary LH surge from an LH baseline has shown great accuracy in locating ovulation but it requires a thorough follicular assessment, more suited to a research setting. A threshold urinary rise in LH to locate the luteal phase has been found here (not shown now) and also in a recent publication (19), to be unreliable on its own (31, 38). These LH in-home tests, could actually help, if associated with mucus peak detection and/or PG rise in-home tests (25, 39, 40; www.mfbfertility.com, 2017], ideally after surpassing the ovulatory threshold during 3 consecutive days, to assure a postovulatory phase (41). Various threshold PG tests could be developed to help in the location, diagnosis and evaluation of the luteal phase. These home tests usually do not consider concentration or hormonal excretion rates, except for the Home Ovarian Monitor assays (42).

### Mucus

Mucus self-perception is yet again shown to be an excellent tool. The BBT shift has revealed to be less accurate than the mucus peak to detect presumed ovulation (17, 18), but it could also be added. Indeed, the mucus peak can be unclear if there is a cervical problem and in some cases of ovulatory dysfunction. It has been found that women can detect their mucus peak with simple written instructions (43). There could even be the possibility for a trained third party to approach the periovulatory period by observing cervical mucus changes. This could be done by adding sequenced vulvar cervicovaginal fluid samples to the urine hormonal samples. This has been done in a previous study with cervicovaginal fluid samples collected from the upper vagina (18), which would not be required. Cervicovaginal fluid samples can also be collected at home in small plastic tubes, after wiping the vulva with a paper tissue. This last approach has been tried successfully, in some cases of difficulty recognizing fertility through mucus perception in a clinical setting, and requires further research. Sequenced cellphone pictures taken by the women of vulvar mucus could also help to locate the luteal phase.

### Luteal profile

Besides classifying by profile in the low tercile, below the 10th percentile, or more strictly below the 5th percentile, the observation of a composite picture of serial EG and PG that can be compared to the described ranges seems clinically sound. Hormonal ranks of the 10th percentile, over the 5th percentile, have been considered here because levels associated with eutrophic term birth have been reported higher than what is usually considered (36). Measuring only PG in the middle luteal zone appears limited as an initial approach. On the other hand, midluteal monitoring of PG and also EG could be enough to guide treatment in most cases, as clinicians currently do in practice (36, 44, 45). Evaluation would best done in at least two cycles, to lower the possibility of assessing isolated poor luteal phases and to consider recurrence. Luteal failure by zone has already been defined (34). All of these have been found here with the ≤ 10th percentile hormonal ranges. More prospective studies in a wider population are needed to associate the decreased hormones of a specific luteal zone with clinical matters.

### Limitations

The reported hormonal analysis would need to be done today through other procedures in a clinical setting. Also the women's fertility status may have changed over time. The possible clinical implications of unrecognized recurrent ovulatory dysfunction and pregnancy outcome after deficient luteal phases that have been discussed herein are far beyond the scope of this study and need much further analysis. These have been highlighted only to stress the potential usefulness of a comprehensive hormonal evaluation of the luteal phase.

## Concluding remarks

A clinically feasible proposal to thoroughly study the luteal phase hormonal profile has been offered. A preliminary range for the main luteal urinary reproductive hormones and a luteal phase classification by zone has been provided. The importance of accurate ovulation detection to correctly locate the luteal phase, in order to evaluate it properly, has been highlighted. Also, different aspects of hormonal urine sample collection and the concentration or excretion rate adjustment have been evaluated. The clinical importance of the assessment of the luteal phase has merely been discussed, and further studies should be conducted on this matter.

## Author contributions

MA conducted the initial study, followed up with the women, designed the secondary study, analyzed and interpreted data, and drafted the manuscript; JA encouraged and contributed intellectually throughout the process, critically revised the manuscript several times and approved the final version; UG and FM studied and tried different algorithms to detect presumed ovulation through urinary LH and PG rise and contributed intellectually in this aspect.

### Conflict of interest statement

The authors declare that the research was conducted in the absence of any commercial or financial relationships that could be construed as a potential conflict of interest.
