# Supplementary material for: Towards the Clinical Evaluation of the Luteal Phase in Fertile Women: A Preliminary Study of Normative Urinary Hormone Profiles
Source: Front Public Health. 2018 May 31;6:147. doi: 10.3389/fpubh.2018.00147 (PMC5991009; doi:10.3389/fpubh.2018.00147)
Supplement: Supplementary file 1 [file Table_1.docx]

**SUPPLEMENT 1**

LH and PG rise determination:

To select the optimum way of determining the LH and PG rise, different methods were applied to this data. The following methods have been considered best to address this matter.

LH baseline: mean LH average of all follicular values before a visual estimation of the LH surge onset (1). Values of visually estimated prepeak surges (2), not close to the PG rise, have been excluded.

LH rise: a rise over the LH baseline of 30% of the LH amplitude (3, 4), close to the PG increase.

LH amplitude: difference between the LH peak and the LH baseline value (3).

LH peak: day with the highest LH numerical value after the LH rise, close to the PG increase.

REFERENCES

1. Godbert, S., Miro, F., Shreeves, C., Gnoth, C., & Johnson, S. (2015). Comparison between the different methods developed for determining the onset of the LH surge in urine during the human menstrual cycle. *Archives of gynecology and obstetrics*, 292 (5), 1153-1161.

doi: 10.1007/s00404-015-3732-z.

1. Alliende M.E. (2002). [Mean versus individual hormonal profiles in the menstrual cycle.](https://www.ncbi.nlm.nih.gov/pubmed/12095496) *Fertil Steril.* 78:90-5.
2. Park S.J., Goldsmith L.T., Skurnick J.H., Wojtczuk A., Weiss G. (2007). [Characteristics of the urinary luteinizing hormone surge in young ovulatory women.](https://www.ncbi.nlm.nih.gov/pubmed/17434509) *Fertil Steril.* 88:684-90.
3. Direito A., Bailly S., Mariani A., Ecochard R. (2013). [Relationships between the luteinizing hormone surge and other characteristics of the menstrual cycle in normally ovulating women.](https://www.ncbi.nlm.nih.gov/pubmed/22999798) *Fertil Steril.* 99:279-85.

doi: 10.1016/j.fertnstert.2012.08.047.

**SUPPLEMENT 2**

Sum luteal PG (or EG): sum of all the luteal PG (or EG). For this issue the few missing samples in this study were assumed as the mean of the value the day before and after.

Mean luteal PG (or EG): average of all the luteal PG (or EG).

Luteal phase zones: according to luteal phase day determined after presumed ovulation.

Initial (in): luteal day 1 to 4.

Medial (med): luteal day 5 to 9.

Final (fin): luteal day 10 onward.

In order to more accurately locate the luteal zone relative to the mucus peak, it seems better to locate the medial zone from the post peak day +5 to +8, and the final zone from day +9 onwards. In our analysis, for the sake of simplification, the same luteal zone days were used after presumed ovulation through LH and mucus peak.

Luteal phase hormonal profile:

“Low” luteal hormone range: sum and/or mean luteal PG or EG ≤ 10^th^ percentile.

“Partially low” luteal hormone range: sum and mean luteal PG or EG > 10^th^ percentile, but in one or more luteal zone/s (initial, medial, final) the mean is ≤ 10^th^ percentile.

≤ Percentile 10: included “Low” and “Partially low” hormone ranges.

Greater than the 10^th^ percentile: sum and mean luteal PG or EG > 10^th^ percentile and in all luteal zones (initial, medial, final) the mean is > 10^th^ percentile.
